# Optimizing Defect Detection on Glossy and Curved Surfaces Using Deep Learning and Advanced Imaging Systems

**DOI:** 10.3390/s25082449

**Published:** 2025-04-13

**Authors:** Joung-Hwan Yoon, Chibuzo Nwabufo Okwuosa, Nnamdi Chukwunweike Aronwora, Jang-Wook Hur

**Affiliations:** Department of Mechanical Engineering (Department of Aeronautics, Mechanical and Electronic Convergence Engineering), Kumoh National Institute of Technology, 61 Daehak-ro, Gumi-si 39177, Gyeonsangbuk-do, Republic of Korea; 20246058@kumoh.ac.kr (J.-H.Y.); okwuosachibuzo3@kumoh.ac.kr (C.N.O.);

**Keywords:** convolutional neural network, ResNet-50, VGG-16, Dijkstra’s algorithm, glossy surface, curved surface, fault classification, fault detection

## Abstract

The industrial application of artificial intelligence (AI) has witnessed outstanding adoption due to its robust efficiency in recent times. Image fault detection and classification have also been implemented industrially for product defect detection, as well as for maintaining standards and optimizing processes using AI. However, there are deep concerns regarding the latency in the performance of AI for fault detection in glossy and curved surface products, due to their nature and reflective surfaces, which hinder the adequate capturing of defective areas using traditional cameras. Consequently, this study presents an enhanced method for curvy and glossy surface image data collection using a Basler vision camera with specialized lighting and KEYENCE displacement sensors, which are used to train deep learning models. Our approach employed image data generated from normal and two defect conditions to train eight deep learning algorithms: four custom convolutional neural networks (CNNs), two variations of VGG-16, and two variations of ResNet-50. The objective was to develop a computationally robust and efficient model by deploying global assessment metrics as evaluation criteria. Our results indicate that a variation of ResNet-50, ResNet-50_224_, demonstrated the best overall efficiency, achieving an accuracy of 97.97%, a loss of 0.1030, and an average training step time of 839 milliseconds. However, in terms of computational efficiency, it was outperformed by one of the custom CNN models, CNN_6_-240, which achieved an accuracy of 95.08%, a loss of 0.2753, and an average step time of 94 milliseconds, making CNN_6_-240 a viable option for computational resource-sensitive environments.

## 1. Introduction

Over the years, the manufacturing sector has undergone a remarkable transformation since the introduction of Industry 4.0, uniquely revolutionizing industrial operations through smart technological implementations. These innovations have enhanced and fostered global innovation and competitiveness within the manufacturing sector. Artificial intelligence (AI) has been used not only in manufacturing processes but also plays a crucial role in the maintenance and reliability of industrial machinery. One aspect of AI, known as Prognostics and Health Management (PHM), ensures uninterrupted production by monitoring the life and performance of a system. PHM enables real-time monitoring and assessment of systems, with the capacity to monitor a system both online (while in operation) and offline (when not in operation) [1]. It can predict the current and future state of a given system based on information generated through sensor technology. Although PHM originated in the aerospace industry, its reach has expanded to industries such as manufacturing, energy, production, automotive, construction, textile, healthcare, and pharmaceuticals.

Vision-based analysis has been successfully employed in manufacturing for standard and quality control inspections [2]. It is also used in technical fields such as intelligent traffic monitoring and unmanned aerial vehicles [3,4]. Despite the success of Prognostics and Health Management (PHM) models in system diagnostics and monitoring, researchers continue to enhance condition-based system models to improve efficiency and robustness, addressing unresolved challenges. One such challenge is automated defect inspection and machine vision-based fault detection for glossy and curvy surfaces. The complex reflectiveness and curvy morphology of these surfaces often cause defects to go unnoticed when captured by traditional cameras [5,6]. For decades, such inspections have been performed manually by trained human inspectors, which is time-consuming and costly compared to automated non-glossy surface inspection [5,7].

Generally, product quality inspection is critical in manufacturing, with methods like visual inspection, automated optical inspection (AOI), machine vision, infrared thermography, laser scanning, and X-ray inspection widely used [8,9,10]. Among these, computer vision stands out for its precision, computational efficiency, and non-contact approach, particularly as industries shift from Industry 4.0 to Industry 5.0 [11,12]. However, issues experienced with glossy and curved surfaces have prompted researchers to explore different methodologies to overcome these challenges.

Some methods for mitigating glossy surface effects primarily involve modifying image capture techniques and using advanced cameras with enhanced lighting to reduce surface reflectiveness. Techniques such as polynomial texture mapping, surface-enhanced ellipsometry, specular holography, and near-field imaging have proven effective when implemented correctly [13,14,15]. Their efficacy is demonstrated in several studies. For example, Müller used polarization filters to diminish surface reflectiveness, capturing two images with different filter orientations and then deriving the specular reflectance intensity on plane surfaces, effectively mitigating surface reflections [16]. Similarly, Yoon et al. [17] proposed a method to remove light reflections in medical diagnostic imaging by adjusting the angle of a linear polarized filter. By controlling vertical and horizontal polarization through filter rotation, they eliminated light reflections and expanded the field of view. In another study [18], the authors introduced a spatial augmented reality framework to improve the appearance of glossy surfaces. Their technique spatially manipulates an environment’s appearance, allowing a glossy surface to reflect a projected image without direct modification, thus enabling effective appearance editing through careful content control. Nevertheless, environmental factors, incompatibility with certain surfaces, and reduced image resolution remain limitations of this approach [19,20,21].

Another unique method often utilized involves implementing advanced algorithms and unique image processing deep learning models, robust enough to handle glossy surfaces. The study by Yuan et al. [22] proposed a dual-mask-guided deep learning model specifically designed to detect surface defects on highly reflective leather materials. Their model enhances defect detection accuracy by effectively removing surface specular highlights in images while preserving bright defects. Although leather surfaces may not be perfectly reflective, this technique could be effective on highly glossy surfaces when implemented with robust models. In another notable study [23], the authors presented an object detection and classification model of polished metal shaft surface defect using a deep learning method based on convolutional neural network feature extraction. Their method was achieved through image segmentation, architecture setup, and parameter optimization of a Fast-R-CNN object detection framework. According to the assessment, their model proved to be implementable in practical production; hence, their model can also be extended to other fields of large image micro-fine defects with large light surfaces. However, model performance can decline in real-life applications involving intense reflections or varying levels of reflectiveness. For example, a glass defect detection method proposed in [20] struggled when the background image closely resembled the reflected image.

For curved surfaces in fault detection—especially with fixed cameras—researchers have developed various techniques to address the challenges. Among these, robotic arms stand out for their flexibility in navigating curved geometries. Wang et al. [24] demonstrated a robotic arm equipped with a 3D micro X-ray fluorescence (µXRF) spectrometer and a depth camera for high-precision scanning of curved surfaces. They emphasized that maintaining consistent X-ray incident angles and scanning distances minimizes counting errors and improves accuracy. The arm’s flexible six-axis design enables comprehensive surface inspections from multiple perspectives, enhancing its industrial utility. Similarly, Huo et al. [25] highlighted the advantages of robotic arms by employing a six-degree-of-freedom manipulator integrated with a line scan camera and high-intensity lighting. This setup successfully inspected convex, free-form, and specular surfaces by dynamically adapting the region of interest, ensuring robust defect detection on complex geometries.

Regardless of the technique used to establish a robust image fault detection or classification model, the classifier or fault detection algorithm remains critical to achieving accurate results. Among deep learning algorithms, convolutional neural networks (CNNs) stand out as one of the most powerful. They have dominated the field of computer vision since their remarkable performance in the ImageNet Large Scale Visual Recognition Competition in 2012 and are widely employed in diverse areas, including medical imaging for tumor detection and fault identification using MRI and X-ray images [26,27,28]. A typical example can be seen in this study by Rajeshkumar et al. [29]; they used CNNs to diagnose brain tumors via MRI scans, emphasizing prompt and efficient defect detection in medical practice. Their methodology demonstrated the flexibility of CNNs; by employing a grid search optimization technique, they enhanced the performance of three CNN models for multi-classification tasks involving brain tumor images. While the models exhibited varying performance levels, they demonstrated the flexibility and adaptability of CNNs. The high accuracy achieved in identifying and classifying brain tumors further underscores the reliability and effectiveness of CNNs in medical image analysis.

Over the years, convolutional neural networks (CNNs) have branched into various specialized architectures to tackle specific challenges in computer vision—including segmentation, object detection, fault detection, and more. For instance, the Visual Geometry Group (VGG) architecture is a standard CNN model with multiple sequential layers, emphasizing simplicity in design to enhance feature extraction [30,31]. However, this comes at a high computational cost due to its depth. The Residual Neural Network (ResNet) architecture revolutionized deep networks by introducing residual connections, which address the vanishing gradient issue and enable much deeper architectures [32].

The strength of ResNet was demonstrated in [33], where several CNN architectures—including Inception V3, VGG-16, VGG-19, and a conventional CNN—were compared for early fault detection in rice leaf blast disease. ResNet-50 achieved a top accuracy of 99.75%, highlighting the importance of deep feature extraction in agricultural applications. In some cases, combining multiple CNN architectures can yield better results. For example, in [34], the authors employed a combination of CNN subtypes for COVID-19 detection and classification. They used Faster R-CNN with a VGG-16 backbone to detect and classify COVID-19 infections in computed tomography (CT) images, achieving an accuracy of 93.86%. This further validates the utility of region-based CNNs in medical image analysis. CNN has also been integrated into various advanced models to create more sophisticated frameworks. For instance, the authors in [35] proposed a novel transformer network enhanced with a CNN cross-attention mechanism for hyperspectral image (HSI) classification. Despite the constraint of limited data samples, their framework demonstrated exceptional classification performance. In another study, He et al. [36] utilized a cross-fusion of CNN and transformer architectures for high-speed railway dropper defect detection. Their robust framework achieved accurate defect detection even under challenging weather conditions, such as rain, fog, sunlight, and nighttime. Deployed across over 300 high-speed trains, their system successfully detected more than 10,000 dropper defects, outperforming most state-of-the-art networks in terms of competence and reliability.

**Figure 1:**
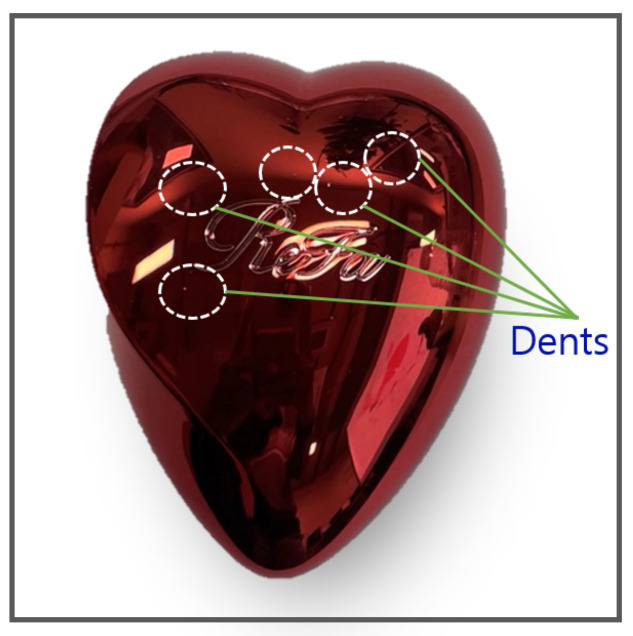
Heart case product sample.

The motivation behind this study stems from the pressing demand within a manufacturing company to create a reliable classification model for products with glossy and curved surfaces. Figure 1 illustrates a product sample: a uniquely designed hairbrush case cover tailored for women. This product merges aesthetic appeal with practical functionality, catering to a specific consumer need. Its distinctiveness arises not only from its visual design but also from its ergonomic construction, material durability, and comfortable palm fit. Beyond its user-centric features, the case enhances the hairbrush’s longevity by providing protection and portability. Its versatile design ensures adaptability to diverse settings and uses, further solidifying its value in everyday life.

Nevertheless, the product’s aesthetic nature not only attracts customers but also poses challenges. Its glossy and curved design complicates traditional machine-vision inspection techniques. For example, as shown in Figure 1, the white circles highlight dent locations that are difficult to detect visually due to surface reflectivity. Quality inspection is critical for this product, as the target demographic demands flawless items, and even minor defects like cracks, dents, or scratches lead to rejection. This stringent requirement has prompted online platforms to automatically flag and reject listings with such imperfections.

To ensure that fault classification is effectively carried out on glossy and curved surfaces in the manufacturing industry, this study makes the following contributions:

- A proposal for integrating a Basler vision camera with enhanced lighting to achieve clear, high-quality image acquisition of glossy surfaces. This integration is aimed to facilitate the development of a robust fault detection and classification framework specifically designed for reflective surfaces.
- The utilization of a KEYENCE laser displacement sensor in combination with a Motoman-GP7 Yaskawa robotic arm to enable the precise and effective acquisition of curved geometries. Additionally, the integration of images from the Basler vision camera and the laser displacement sensor is proposed as an ideal approach for developing a fault classification framework for glossy and curved surface image products.
- A thorough comparative assessment of robust deep learning algorithms for image-based fault detection, with global assessment metrics such as accuracy, loss, computational efficiency, recall, F1 score, specificity, mAP, and confusion matrix, to validate the performance of our model and identify an efficient algorithm that is also computationally cost efficient.

The rest of the paper is structured as follows: Section 2 explains the theoretical background of the core techniques employed in the study. Section 3 details the proposed system framework and methodologies used to achieve the study’s objectives. Section 4 presents the experimental setup and visualizations. Section 5 discusses and evaluates the results, while Section 6 concludes the study.

## 2. Theoretical Background

This section discusses the theoretical background of CNN architecture and its sub-types as employed in the study. It also explores key techniques utilized in the development of the proposed framework.

### 2.1. Convolutional Neural Network

Convolutional neural networks (CNNs), or ConvNets, are a class of deep learning models with multi-layered neural network architectures. They excel in tasks such as image classification, object detection, segmentation, natural language processing, and time series analysis, and are widely used in fields like computer vision and fault detection [37,38]. Their ability to learn directly from raw data, eliminating manual feature extraction, enhances their efficiency in complex tasks with minimal human intervention.

First conceptualized by Hubel and Wiesel in the 1950s–1960s through studies of the visual cortex, CNNs gained prominence in 2012 after dominating the ImageNet Large Scale Visual Recognition Competition [37,39]. CNNs are categorized by input dimensionality: 1D CNNs are used for sequential data such as audio and time series, 2D CNNs excel in image-related tasks like classification and segmentation, and 3D CNNs handle volumetric datasets such as CT scans and video [40,41,42]. Each category employs specialized kernels to extract task-specific features, making CNNs versatile for applications like fault detection and signal processing.

#### CNN Architectural Overview

A typical CNN architecture is composed primarily of six layers, namely:

- Input layer;
- Convolutional layer;
- Pooling layer;
- Flatten layer;
- Fully connected layer;
- Output layer.

A typical convolutional neural network (CNN) consists of specialized layers, each performing distinct tasks, making it a powerful algorithm for image understanding tasks such as processing, classification, and fault detection. The architecture begins with the input layer, which determines the nature and format of the input. For image inputs, pixels represent numerical color grades (0–255), forming a matrix that the CNN processes. The brightness, shade, and tint of an image are determined by pixel value grading, which is sequentially organized into a digital layout [37,38]. The input layer is critical as it defines the raw data structure, influencing model performance. Factors such as image resolution, data augmentation, normalization, and input channels (e.g., grayscale, RGB, or RGBA) must align with the input layer format to ensure optimal performance [43,44]. Normalization scales pixel values from 0–255 to 0–1, ensuring dataset consistency and training stability. Mathematically, normalization is expressed as follows:

(1) $$I_{norm}\left(x,y,c\right)=\frac{I(x,y,c)}{255}$$

where $I_{norm}$ stands for the normalized image pixel value, *I* presents the original pixel value. The output of the input layer, a tensor, is passed to the convolutional layer for feature extraction. This layer uses filters (kernels), small matrices of learnable weights, to convolve through the input array, producing feature maps [45]. The convolution operation is defined as follows:

(2) $$f_{ij}^{k}=\sum_{u=1}^{U}\sum_{v=1}^{V}\sum_{c=1}^{C}I_{(i+u)(j+v)c}\;\cdot \;K_{uvc}^{k}+b^{k}$$

where $f_{ij}^{k}$ stands for the output values at positions *i* and *j* in the *k*-th feature map. *U*, *V*, and *V* represent the filter’s height, width, and input channels, respectively; $K_{uvc}^{k}$ stands for the filter weight at positions *u* and *v* for the *c*-th channel of the *k*-th filter; and $b^{k}$ is bias.

Filter size (e.g., 3 × 3, 5 × 5, or 7 × 7) determines the level of detail captured, with smaller filters extracting finer details and larger filters capturing broader context. Convolutional layers are stacked to progressively extract low-level (edges, corners), mid-level (shapes, patterns), and high-level (complex patterns) features, enabling hierarchical representation learning [46]. The number of filters in a layer determines the variety of features learned; for example, 16 filters generate 16 feature maps. Strides and padding further influence the output: strides control the filter’s step size (e.g., stride 1 processes all pixels), while padding (e.g., Same or Valid) determines whether input edges are included in computations, affecting output dimensions [43,47].

The pooling layer, also known as downsampling, refines the features extracted by the convolutional layer by reducing the dimensionality of the feature map while retaining the most significant features. This process enhances the network’s performance by maintaining accuracy and computational efficiency [37,43]. Pooling operations include max pooling, average pooling, and global pooling. Max pooling selects the maximum value within a pooling window, while average pooling computes the average value in the window. Global pooling applies max or average pooling over the entire feature map, reducing it to a single value per channel [37,48].

The pooling window size is a critical parameter, as it determines the region over which the pooling operation is applied and influences the nature of downsampling. Smaller window sizes, such as 2 × 2, preserve finer details in the feature map, whereas larger windows, like 3 × 3, provide more aggressive dimensionality reduction, potentially sacrificing fine details but capturing broader contextual features. The stride, which controls the movement of the pooling window, also plays a significant role. Larger strides result in greater dimensionality reduction, while smaller strides retain more spatial details in the output feature map. Mathematically, the output dimensions of the pooling layer can be expressed as follows:

(3) $$O_{d}=\frac{I_{d}-T_{d}+2P}{S}+1$$

where $O_{d}$ represents the output and its dimensions; $I_{d}$ represents the input and its dimensions; and $T_{d}$ stands for the pooling window and its dimensions. *P* and *S* represent padding and strides, respectively.

Additionally, the operations for max pooling and average pooling are defined as follows:

(4) $$P_{ij}=\max\left(I_{\left(i:i+h-1,j:j+w-1\right)}\right)$$

(5) $$P_{ij}=\frac{1}{h\;\cdot \;w}\sum_{x=i}^{i+h-1}\sum_{y=j}^{j+w-1}I_{xy}$$

Generally, the pooling layer’s effectiveness depends on the careful selection of window size and stride, which together determine the balance between dimensionality reduction and feature retention [37,43,48].

The flatten layer serves as a critical transition between the convolutional/pooling layers and the fully connected layer, reshaping the multidimensional feature maps from the pooling layer into a one-dimensional vector. This transformation ensures that spatial features extracted by earlier layers are preserved and formatted for processing in the fully connected layer [49,50]. For instance, a pooling layer output of size 3 × 3 with 64 feature maps is flattened into a vector of size 576 (3 × 3 × 64). Once flattened, the data is passed to the fully connected layer, where every neuron connects to every neuron in the previous layer, enabling dense connectivity. This layer aggregates the extracted features and processes them for tasks such as classification, regression, or prediction [37,43]. Each neuron in the fully connected layer computes a weighted sum of its inputs, adds a bias, and applies an activation function to introduce non-linearity, as described in Equation (6):

(6) $$F=\sigma \left(\sum_{i=1}^{n}W_{i}x_{i}+b\right)$$

where *F* is the output of the neuron; σ is the activation function; $\sum_{i=1}^{n}$ represents the summation of all input; and $W_{i}$ is the weight of the *i*-th input. $x_{i}$ and *b* represent the input vector and bias, respectively.

The activation function plays a vital role in both convolutional and fully connected layers, enabling neurons to detect complex patterns and hierarchies in the input data. In convolutional layers, activation functions process and retain spatial features extracted by filters, while in fully connected layers, they introduce non-linearity, allowing the network to learn and understand the complexity of the data. Without activation functions, neurons would only perform linear transformations, limiting the network’s ability to model intricate relationships [51,52]. Commonly used activation functions include ReLU, Softmax, and Sigmoid, each serving specific purposes; these are represented mathematically in Equation Equations (7)–(9) below.

(7) ReLU(x)=max(0,x)

(8) $$Softmax\left(x_{i}\right)=\frac{e^{x_{i}}}{\sum_{j=1}^{n}e^{x_{j}}}$$

(9) $$Sigmoid\left(x\right)=\frac{1}{1+e^{-x}}$$

where *x* is the input and *e* represents Euler’s number.

ReLU is widely used in intermediate layers to prevent vanishing gradients and ensure efficient training, as it activates neurons only for positive inputs. Softmax is typically employed in multi-class classification tasks, converting outputs into probabilities, while Sigmoid is used for binary classification, mapping outputs to a range between 0 and 1. The choice of activation function depends on the task and defines the output layer of the CNN architecture [37,53].

Despite their effectiveness, fully connected layers have limitations, such as high computational costs and susceptibility to overfitting. These challenges can be mitigated through proper parameter tuning, including the selection of optimal layer sizes, filters, padding, and strides, as well as the use of regularization techniques. Regularization methods—such as batch normalization, L1/L2 regularization, early stopping, and dropout—improve a model’s ability to generalize to unseen data by adding constraints that prevent overfitting [54]. Batch normalization stabilizes the learning process by normalizing inputs to each layer, while L1 regularization encourages sparsity by penalizing the absolute values of weights. L2 regularization, on the other hand, penalizes squared weights, ensuring even weight distribution without driving them to zero. Elastic Net combines L1 and L2 penalties, balancing sparsity and weight stability [37,43,54]. Early stopping monitors validation performance during training and halts the process when improvements plateau, while dropout randomly deactivates neurons during training, forcing the model to learn robust and redundant features. Additionally, data augmentation is a powerful technique to combat overfitting, especially with limited datasets. It generates additional training samples by applying transformations such as zooming, flipping, and adjusting brightness or intensity. These transformations increase data variability and complexity, ensuring the model generalizes better to real-world variations, regardless of dataset size [37].

### 2.2. Visual Geometry Group 16 Layer CNN (VGG-16)

VGG-16 is one of the most popular CNN architectures, developed by the Visual Geometry Group (VGG) at the University of Oxford. It was first implemented in 2014 by Simonyan and Zisserman for large-scale image recognition, as described in their study [55]. The name VGG-16 comes from the number of trainable layers in the network, which totals sixteen trainable layers comprising thirteen convolutional layers and three fully connected layers. Although the network contains a total of twenty-one layers, some of these are non-trainable (e.g., pooling layers).

The architecture was designed to deepen the structure of traditional CNNs while maintaining simplicity in the network design. One of its standout features is the use of 3 × 3 convolutional kernels, which help capture local patterns in the input data while maintaining computational efficiency. To further increase its depth, VGG-16 utilizes a stack of these small filters, enabling the model to train on and learn more complex features. Another key feature of VGG-16 is the application of max-pooling layers after every 2–3 convolutional layers. These pooling layers use a 2 × 2 filter size with a stride of 2 to reduce the spatial dimensions of the feature maps, making the model computationally efficient while preserving the most important features. At the end of the convolutional stack, the network outputs a feature map passed through fully connected layers, followed by a Softmax layer for multi-class classification. However, though it is highly efficient for feature extraction and transfer learning, VGG-16 is notable for being more computationally demanding than traditional CNN due to its large number of parameters [56]. For instance, it has two dense layers with 4096 neurons each, with an output layer of 1000 neurons in the case of Imagenet classification, and it has a total of 138 million parameters.

### 2.3. Residual Network (ResNet)

ResNet is a distinctive CNN architecture first introduced by He et al. in their study [32]. It was specifically designed to address the vanishing gradient problem, a common issue in deep neural networks where gradients become increasingly small during backpropagation, hindering effective learning. This problem is particularly evident in architectures like VGG-16. ResNet resolves this issue through residual learning and skip connections, which ensure smoother gradient flow and mitigate the vanishing gradient problem. As the name suggests, ResNet consists of various layers, including convolutional layers, activation functions, normalization layers, and bottleneck residual blocks [32].

The skip connection and bottleneck residual block are key characteristics of ResNet-50 that enable it to achieve high efficiency and lower computational costs compared to similar CNN architectures. Skip connections allow the input to bypass certain layers and be added directly to the output, enabling the network to learn residual functions. These residual functions are generally easier to optimize than direct mappings. On the other hand, the bottleneck structure, which uses 1 × 1 convolutions to reduce and then restore dimensionality, ensures smoother gradient flow during backpropagation, reducing computational requirements [32].

ResNet architectures come in various depths, with the most popular models being ResNet-18, ResNet-50, ResNet-101, and ResNet-152, each differing in the number of layers. Due to its unique architecture, ResNet—particularly ResNet-50—has been successfully employed in a variety of fields due to its efficiency and robust performance in image classification, object detection, and numerous computer vision tasks [32,33].

### 2.4. Dijkstra’s Algorithm Overview

Dijkstra’s algorithm is one of the popular algorithms introduced by Edsger W. Dijkstra that can be employed to determine the shortest paths in weighted graph instances where edges have non-negative weights [57,58]. This technique, known for its simplicity and efficiency, has been widely used in solving graph-based shortest path problems and has found applications in various domains such as GPS navigation, network routing, and optimization problems. The core principle of Dijkstra’s algorithm works by exploring the paths from nodes to the source node, selecting the shortest possible path among them. Dijkstra’s algorithm employs a greedy approach, enabling it to make a locally optimal choice at every step, aiming to achieve the global optimum [57,58]. The algorithm begins by initializing all nodes with a tentative distance of infinity, except for the source node. This process is repeated until the shortest distance is determined. In practice, the initial distance of the source node is set to zero, and the other nodes are set to infinity. The algorithm marks all nodes as unvisited and, for each unvisited neighbor, calculates their tentative distances by adding the weight of the edge connecting the current node to the neighbor. Once all neighbors of the current node are processed, the node is marked as visited, and the algorithm moves to the next unvisited node with the smallest tentative distance. This process is repeated until all nodes have been visited, or the remaining unvisited nodes are not connected to the source. As the algorithm terminates, each node will have the shortest distance from the source node, providing the solution to the problem.

In the context of graph theory, given that G=(V,E) where *V* and *E* represent the vertices and edges of a given graph, and assuming each edge (u,v) has a weight of w(u,v), then d[v] stands for the shortest distance from a source node *s* to the node *v*, and p[v] represents the previous node in the shortest path from the source node *s* to node *v*. Dijkstra’s algorithm initializes using Equation (10).

(10) Set:d[s]=0,d[v]=∞forallv≠s

where *u* is the current node and *v* is the neighbor node.

At this point, all nodes are marked as unvisited. While the nodes are unvisited, each neighboring node *v*, updates its distance using Equation (11):

(11) d[v]=min(d[v],d[u]+w(u,v))

After this, node *n* is marked as visited, and the algorithm moves to the next unvisited node with the shortest distance of d[v].

## 3. Proposed System Framework and Methodology

**Figure 2:**
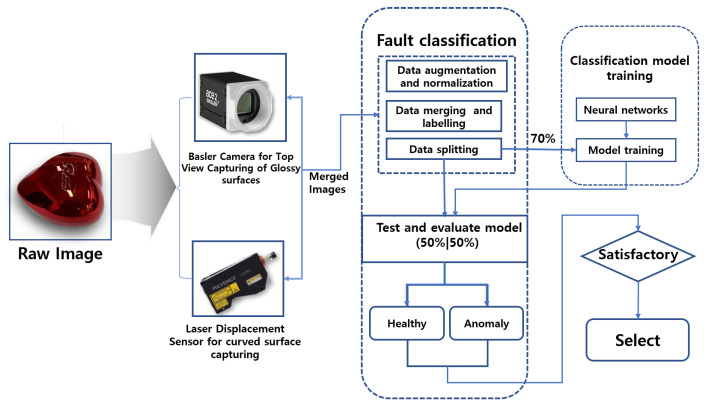
Proposed system framework.

The proposed framework, illustrated in Figure 2, outlines the steps and methodologies used in our study to achieve fault diagnostic classification for glossy and curved surfaces. A heart-shaped brush case with a glossy and curved surface was selected as the reference sample.

Our methodology consisted of three key steps. The first step involved image data collection, where two robust pieces of equipment were used to ensure the datasets were suitable for deep learning-based fault classification. A Basler vision camera with lighting was employed to capture images of the flat top surface, while a KEYENCE laser displacement sensor, mounted on a robotic arm, was used to efficiently scan the curved surface. The second step focused on data processing, which included data merging, data augmentation, labeling, and dataset splitting. In the final step, the processed datasets were fed into various deep learning image classification models. Validation assessments were conducted to identify the best-performing model and ensure it met the required performance standards.

These stages are implemented to ensure that issues encountered with glossy and curved surfaces are mitigated, thereby providing a reliable framework capable of achieving the desired efficiency for overall model validation. Given the nature of our dataset, which originates from glossy and curved surfaces, we applied specific data augmentation techniques to further enhance the images and ensure optimal adaptation.

### 3.1. Data Augmentation

The primary aim of data augmentation is to introduce variability into the dataset, simulating real-world instances such as zoom, lighting changes, and flipping. This ensures that the model does not rely on overly simplistic patterns, which could lead to overfitting. Data augmentation is particularly crucial when working with unbalanced or small datasets, as it serves as an efficient way to enhance and increase training data without the need to acquire additional samples. In our study, the augmentation techniques employed were brightness, zoom, and custom pixel multiplication, and their mathematical representation is presented thus:

Brightness:

(12) $$\hat{X}\left(i,j\right)=X\left(i,j\right)+\Delta b$$

where (i,j) stands for the pixel value at position (i,j), Δb is the brightness adjustment constant.

Zoom:

(13) $$\;\hat{X}\left(i^{\prime },j^{\prime }\right)=X\left(\frac{i}{Z_{s}},\frac{j}{Z_{s}}\right)$$

$Z_{s}$ stands for the zoom factor.

Contrast:

(14) $$\;\hat{X}\left(i,j\right)=\alpha \;\cdot \;\left(X\left(i,j\right)-\mu_{X}\right)+\mu_{X}$$

Custom Pixel Multiplication:

(15) $$\;\hat{X}\left(i,j\right)=k\;\cdot \;X\left(i,j\right)$$

where *k* is the scaling factor for each pixel value.

In this study, brightness and contrast were adjusted by ±30% and ±20%, respectively, while zoom was adjusted by ±10%. Additionally, a random multiplier of 3.0 was applied.

### 3.2. Model Performance Evaluation Criteria

Model evaluation is important to ensure that the model performs up to a given standard. In this study, these metrics were implemented to help evaluate, validate, and determine the best-performing deep neural network for our framework. Thus, some of the core global performance metrics implemented in this study include accuracy ($A_{cc}$), precision ($P_{rc}$), F1-score ($F_{sc}$), sensitivity ($S_{nv}$), specificity ($S_{pc}$), and mean average precision (mAP), and their mathematical representations are shown in (16)–(20).

(16) $$A_{cc}=\frac{TP}{TP+FP+TN+FN}$$

(17) $$P_{rc}=\frac{TP}{TP+FP}$$

(18) $$F_{sc}=\frac{2*\mathrm{Sensitivity}*\mathrm{Precision}}{\mathrm{Precision}+\mathrm{sensitivity}}$$

(19) $$S_{nv}=\frac{TP}{TP+FN}$$

(20) $$S_{pc}=\frac{TN}{TN+FP}$$

(21) $$\mathrm{mAP}=\frac{1}{N}\sum_{x=1}^{N}AP_{x}$$

TP, FP, TN, and FN stand for true positive, false positive, true negative, and false negative, respectively. $AP_{x}$ represents the average precision of class *x*, while *N* is the total number of classes.

TP represents the number of positive samples that a model accurately classified as belonging to the positive class, while FP indicates the number of non-positive samples that the model falsely classified as belonging to the positive class. On the other hand, TN represents the number of negative samples that the model accurately classified as belonging to the negative class, and FN stands for the number of negative samples that the model falsely classified as belonging to the positive class.

mAP measures the accuracy of a model in classifying within a dataset. It evaluates the model’s performance across all classes and provides a single score for overall assessment, indicating the model’s quality. Furthermore, a confusion matrix was implemented in our study to provide a detailed analysis of the actual classification performance, represented in percentages with respect to TP, FP, TN, and FN.

## 4. Experimental Setup and Visualization

The experimental data collection was conducted at the Defense Reliability Laboratory of the National Institute of Technology Kumoh, Gumi, Republic of Korea. The procedure involved two stages. In the first stage, high-vision imaging combined with appropriate lighting was used to capture a detailed front view of the image data. In the second stage, a laser displacement sensor mounted on a robotic arm was utilized to scan and reconstruct 3D surface data of the curved sections of the dataset.

### 4.1. Data Collection Using Basler Vision Camera

**Figure 3:**
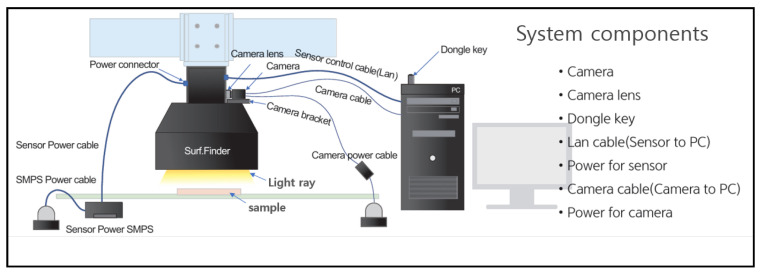
Basler vision and lighting usage overview.

The Basler vision camera equipped with lighting was initially employed to generate image data from our samples in this study. The setup for image data collection using the Basler vision camera and lighting is shown in Figure 3. This setup was used exclusively to capture the front glossy view of the heart-case samples, as it was unable to capture the curved glossy portions of the samples. The curved surfaces were instead captured using laser displacement sensors.

Due to the glossy nature of the heart case samples, a lighting device, Surf.Finder-SF, was introduced to minimize glare and enhance image quality. Table 1 and Table 2 present the specifications of the camera and lighting equipment used. Figure 3 illustrates the schematic diagram of the Basler vision camera setup with the lighting system. When the lighting equipment is powered on through the sensor power SMPS, the Surf.Finder light activates. This lighting system includes adjustable settings to reduce glare, ensuring optimal illumination for the heart case samples. These settings can be modified through a dedicated program, which requires a dongle key to be connected to the computer for access. The camera is powered separately via the camera power SMPS. Focus adjustments can be made using a screw attached to the camera lens. The camera is connected to the PC through a dedicated cable, enabling parameter adjustments and facilitating the transmission of captured images to the CPU for processing, storage, and further analysis.

Three core input parameters implemented include reference (REF), roughness (RGH), and raw gray vertical (RGV).

- REF: This parameter sets the lighting to capture photos by projecting light in all directions. Three shots are taken at 1 ms intervals to minimize reflections caused by brightness.
- RGH: This parameter configures the lighting to check horizontal roughness. Lighting is directed at a 45-degree angle to the vertical direction, and three shots are taken at 1 ms intervals to assess horizontal roughness or changes.
- RGV: This parameter configures the lighting to check vertical roughness. Lighting is set at a 45-degree angle to the horizontal direction, and three shots are taken at 1 ms intervals to assess vertical roughness or changes.

The Surf.Finder lighting system is designed to operate in a controlled environment, minimizing the impact of external lighting variations. Its enclosed casing ensures a consistent illumination field, reducing interference from ambient light sources. Additionally, the adjustability of the REF, RGH, and RGV parameters allows for fine-tuned lighting conditions that adapt to different surface reflectivities, ensuring robust and high-quality image acquisition under varying conditions.

These three setups were used to capture our samples, which were then transferred to a PC for review and selection of the desired data samples for implementation.

### 4.2. Data Collection Using KEYENCE Laser Displacement Sensor

**Figure 4:**
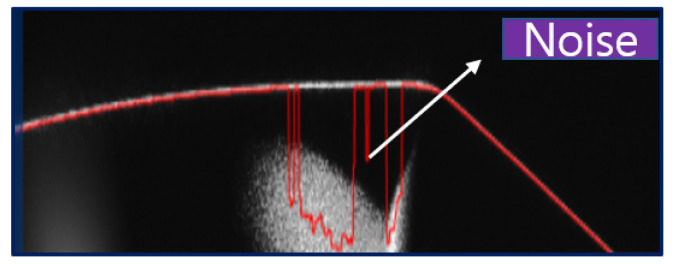
Laser noise overview.

In our setup, we employed a Basler vision camera with controlled lighting to capture front-view image data samples. However, the curved surfaces of the data samples posed challenges for the Basler camera, as its point of view (POV) was not optimal for effectively imaging curved geometries. To overcome this, a laser displacement sensor was introduced. For curved surfaces with glossy finishes, using the laser displacement sensor alone introduced noise due to the diffuse reflection of light during surface scanning, as illustrated in Figure 4. To mitigate this issue, a YASKAWA robotic arm was utilized to scan the surface in a curved motion with the laser displacement sensor. Table 3 summarizes the specifications of the laser displacement sensor used in this study.

**Figure 5:**
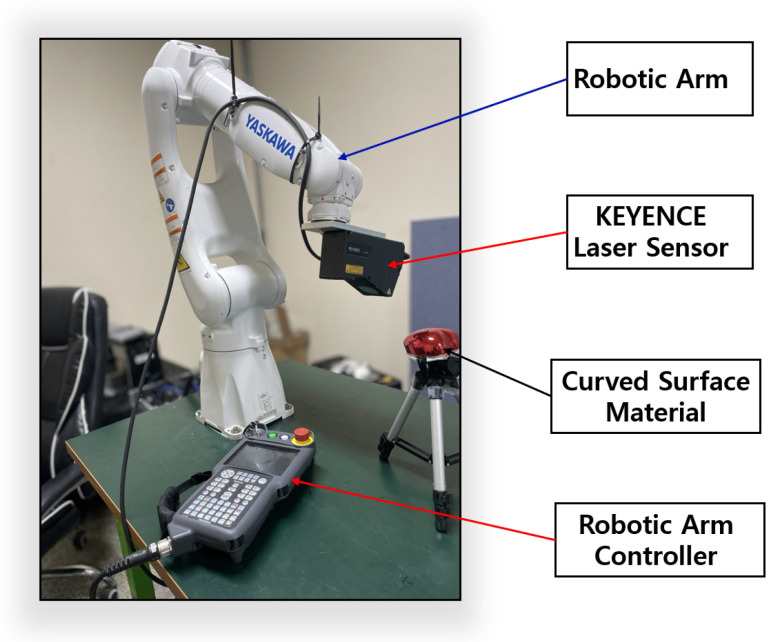
Robotic arm.

Traditionally, visual inspection is performed at least three times for the appearance inspection of glossy products, with an average inspection time of about 15 s. However, to measure a defect of 0.1 mm or more on a curved surface, it takes an average of 60 s for a laser displacement sensor with a precision of about 12.5 μm, operating at a speed of 1.15 cm per second, to scan the entire shape. Therefore, it is necessary to maintain a time period similar to that of visual inspection to avoid disrupting production speed. To address this issue, a vision camera is first used to quickly photograph the front part of the heart case, which has many flat areas, and a laser displacement sensor is used for the side parts to reduce the overall measurement time. The more laser displacement sensors there are, the shorter the measurement time, but the higher the initial cost purchase of the robotic arm and laser displacement sensors. To solve this, the study developed a technology that moves the optimal path at an angle that minimizes reflection using one robotic arm and one laser displacement sensor. Our goal was to achieve an average inspection time of about 15 s. An overview of the setup is shown in Figure 5.

#### Data Generation Using the Laser Displacement Sensor and Robotic Arm

**Figure 6:**
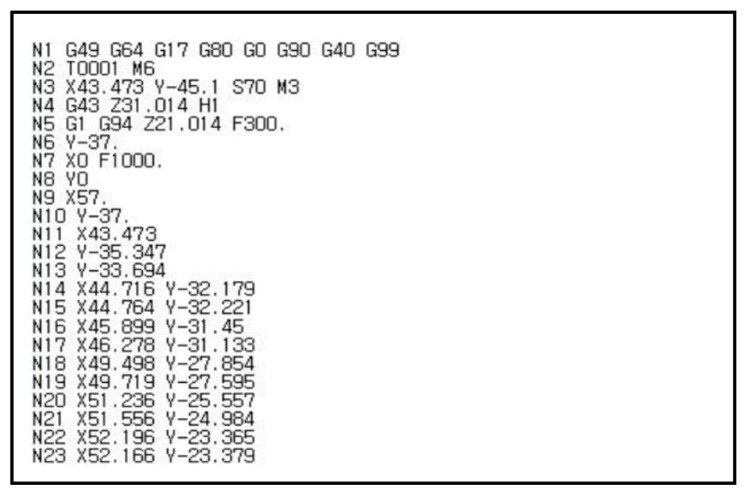
Coordinates obtained through NC module.

To minimize glare caused by the glossy surface of the materials, the primary objective was to determine the normal vectors of all sample surfaces. This was achieved by aligning the vertical relationship between the surface of the heart case samples and the measurement setup, thereby reducing light reflection. To obtain the normal vector coordinates of the heart case-shaped samples, CATIA’s NC (Numerical Control Machining) module was utilized. This module automatically generates tool movement paths and optimizes the machining process by preventing collisions between the tool and the machined object. This optimization is based on the shape of the designed 3D model. Using the NC module, coordinates offset from the surface of the heart case were obtained. To ensure accurate measurement, the laser displacement sensor was set to an identification distance of 70 mm, meaning coordinates were collected 70 mm vertically above the heart case surface. Figure 6 provides an overview of the obtained coordinates.

The collected coordinates represent areas that theoretically minimize diffuse reflection. However, many of these coordinates included overlapping measurement regions. To address this, the teaching function of YASKAWA—which refers to a mode allowing the operator to define tasks, movements, or paths for the robot to follow and execute—was utilized to first identify and select the coordinates that minimized diffuse reflection. Next, overlapping areas among the selected coordinates were eliminated, and the remaining coordinates were further refined through a second selection process. Using the coordinates generated through the NC module and the process of minimizing diffuse reflection while removing duplicates, 10 measurement paths for the sides of the heart case were created. The inspection results, as shown in Table 4, indicate a measurement time ranging from approximately 20 to 26 s.

Since the selected coordinates include several paths, the shortest optimal path among them must be determined. However, before introducing the Dijkstra algorithm, the coordinates of the heart case were selected by manually choosing the areas with the least light reflection on the curved surface from the coordinates extracted through the NC code. The coordinates were selected at 2 cm intervals. In the case of the inspection time, the left side of the heart case was photographed in the order of the front, right side, and back. It took 21 s based on the 11 mm/s movement of the Yaskawa robotic arm that received the coordinates and moved. This was because there were many instances where the same scene was filmed repeatedly, as the focus was on minimizing diffuse reflection. By excluding the overlapping areas through the Dijkstra algorithm, the result was reduced to 15 s based on the 11 mm/s movement. It is observed that the movement path was optimized while excluding overlapping filming scenes. For this purpose, the Dijkstra algorithm was used to determine the shortest path the robotic arm could follow for fast execution. The input data were obtained by calculating the travel time for each coordinate using the 3D coordinates, and one path was selected through the algorithm. The information about the path is summarized in Table 5 below.

The inspection speed is set to 11.5 mm/s because the amount of data collected per unit of time is fixed due to the characteristics of the laser displacement sensor. The amount of data collected during measurement is inversely proportional to the range to be measured. Thus, if the inspection speed exceeds 11.5 mm/s, the shape to be measured is compressed, and if the inspection speed is slower than 11.5 mm/s, the shape to be measured is expanded. Therefore, to measure the heart case shape without distortion, the inspection speed must be set to 11.5 mm/s.

**Figure 7:**
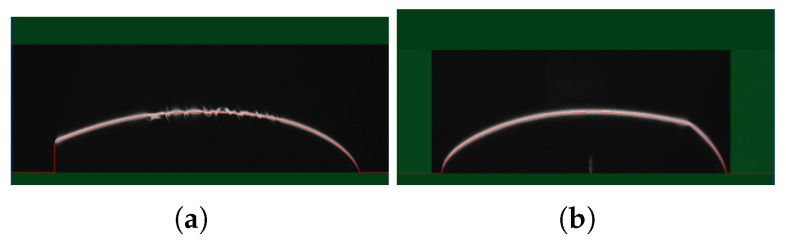
Laser displacement sensor measurable range based on (**a**) data collection speed of 500 Hz; (**b**) data collection speed of 750 Hz.

To match the inspection time to 15 s, a method is required to increase the inspection speed while minimizing distortion in the results. To achieve this, images were captured under conditions where the heart-shaped case measured 90 mm × 90 mm, ensuring that the side could be fully observed in a single pass during measurement. This approach increases the inspection speed by reducing the collection area while simultaneously increasing the amount of data collected within a confined space. As a result, the inspection speed improved from 11.1 mm/s at 500 Hz to 13.2 mm/s at 750 Hz, as shown in Figure 7. The results are summarized in Table 6.

Using the 2D CSV file acquired through a laser displacement sensor, a 2D matrix is generated based on the sensor’s movement trajectory and measurement conditions. The horizontal axis is defined as *x*, the vertical axis as *y*, and the measured values as *z*. The generated matrix is expressed by the following function:

(22) z(x,y)=Resultsfromheightmeasurement

There may be unmeasured areas within the data collection range, and these gaps are filled using interpolation methods such as linear interpolation or spline interpolation. Through this process, a continuous 3D data structure is formed. Subsequently, the matrix is input into 3D graphics software or a visualization tool to generate a 3D surface image. During rendering, tools such as OpenGL 4.6, Matplotlib 3.7.1, or other 3D libraries are utilized to visualize the data in three dimensions.

### 4.3. Dataset Description

**Figure 8:**
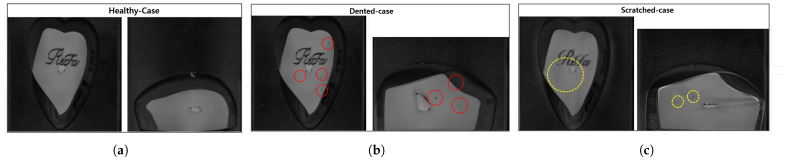
Combined image class samples: (**a**) Healthy class; (**b**) Dent class showing dent portion in red circles; (**c**) Scratch class showing scratch portion in yellow circles.

As previously highlighted, the study utilized two data acquisition techniques: a Basler vision camera with lighting and a laser displacement sensor. Each technique generated two image samples per data point—one from the camera and one from the laser sensor. Data labeling and model selection in this study were guided by customer requirements, including alignment with historical rejection patterns and a target accuracy of 95% or higher. The dataset, derived from production records, was labeled to reflect defect types and severity levels critical to the customer’s quality standards, ensuring the model was trained on operationally meaningful data. The data samples were categorized into three classes, as summarized in Table 7. As a result, six distinct data classes were generated from these three classes using the two techniques: HCC Basler camera images, HCC laser sensor images, DC Basler camera images, DCC laser sensor images, SCC Basler camera images, and SCC laser sensor images. To ensure consistency and reduce data complexity, similar data classes were merged. For instance, HCC Basler camera images and HCC laser sensor images were combined into a single HCC dataset. Pictorial samples of the merged images are shown in Figure 8.

After the combination, a total of 6902 images—2452 HCC images, 2414 DCC images, and 2037 SCC images—were generated, approximately in a ratio of 35.52%, 34.97%, and 29.51%, respectively. In terms of dataset distribution, the data were relatively balanced across categories. However, to mitigate potential bias due to slight class size differences, data augmentation techniques such as brightness adjustment, zooming, contrast enhancement, and custom pixel multiplication—as discussed in Section 3.1—were applied to enhance model generalization. This approach ensured that the model learned meaningful features from all defect types effectively while preventing overfitting to dominant categories.

### 4.4. Deep Learning Algorithms Parameters

The performance of any fault classification model largely depends on the choice of classifier algorithm. To address this, we evaluated four custom CNN architectures alongside four pre-trained models: two variants of VGG-16 and two variants of ResNet-50. Our objective was to develop an efficient model optimized for low computational energy consumption. To achieve this, we designed streamlined architectures that balance performance and efficiency, prioritizing minimal resource usage without compromising accuracy. Additionally, the hyperparameters of the deep learning algorithms were optimized empirically. A summary of the models is provided in Table 8.

#### 4.4.1. Custom CNN Parameters

**Figure 9:**
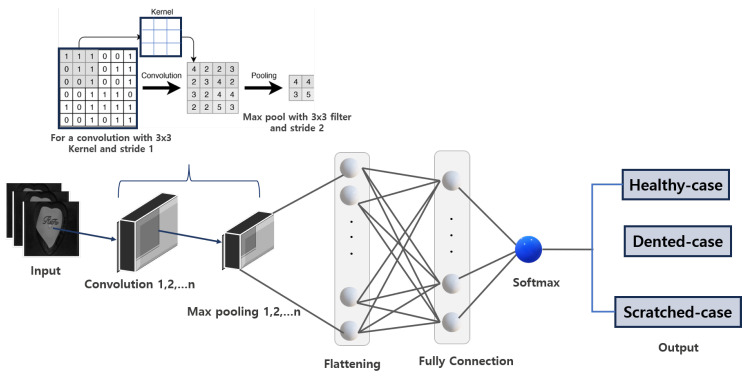
The n-layer structure of the custom CNN architecture with 3 × 3 filter and a stride of 2.

The study evaluated four custom CNN architectures—CNN_5_-128, CNN_5_-240, CNN_6_-128, and CNN_6_-240—each subjected to varying hyperparameter configurations, including the number of convolutional layers, strides, pooling layers, and fully connected layers. These configurations were optimized to evaluate fault classification performance while minimizing computational demands. A summary of the architecture details and the n-layer structure of the custom CNN framework is presented in Table 9 and Figure 9.

The custom CNNs with three convolutional layers (CNN_5_-128 and CNN_5_-240) include 32, 64, and 128 kernels across their respective layers, while the custom CNNs with four convolutional layers (CNN_6_-128 and CNN_6_-240) contain 16, 32, 64, and 128 kernels. All custom CNN models also feature dense layers with 128 kernels. To optimize efficiency and minimize computational energy, the input image dimensions were carefully chosen, as they significantly affect the speed and resource requirements of the CNN models. Common input dimensions for CNNs include 128 × 128, 224 × 224, 256 × 256, or their multiples. While larger dimensions can improve performance by offering more detailed images, they also increase the computational resource demands.

Consequently, we explored image pixel sizes of 128 × 128 and 240 × 240 during training. Dimensions of 256 × 256 and larger were avoided due to their high computational demands. However, we chose 240 × 240 as it provided better image clarity than 224 × 224 while maintaining reasonable computational requirements.

#### 4.4.2. Other Deep Learning Models’ Parameters

VGG-16 was introduced in this study due to its compatibility with our dataset, despite not being the most advanced architecture compared to robust image classification models like Inception, ResNet, and EfficientNet. Although the optimal input size for VGG-16 is typically 224 × 224, we trained VGG-16 variants using two image dimensions: 128 × 128 (VGG-16_128_) and 224 × 224 (VGG-16_224_) to evaluate the best balance between performance and computational requirements. This approach aimed to assess whether comparable results could be achieved with a lower resolution.

Similarly, ResNet-50 was included to ensure the selection of the most suitable neural network for the dataset through performance comparisons. Known for its efficiency and lower computational demands compared to VGG-16, ResNet-50 was chosen to provide a practical assessment of model performance, rather than relying solely on assumptions. Although ResNet-50 often outperforms VGG-16 in many applications, the effectiveness of any model depends heavily on its adaptability to the specific dataset. To further optimize ResNet-50 for low computational energy, we trained it using two image dimensions: 128 × 128 (ResNet-50_128_) and 224 × 224 (ResNet-50_224_) to identify the best performing configuration. A summary of the VGG-16 and ResNet-50 architectures used in this study is provided in Table 10.

The models were executed with unfrozen layers to achieve the desired results. This approach allows for weight updates during the training process, enabling the model to adapt to the specific dataset.

## 5. Result Evaluation and Discussion

The eight neural networks were empirically evaluated and assessed using three image classes, with the evaluation based on global metrics outlined in the previous section. This approach ensured the selection of the best model for our fault classifier. In addition to performance metrics, computational cost and the confusion matrix were also considered to guarantee a comprehensive evaluation of the models. A total of 6902 images were used, consisting of 2452 HHC, 2414 DCC, and 2037 SCC images. The dataset was divided into training, validation, and testing sets. For VGG-16 and ResNet-50, a 70:15:15 ratio (training:validation:testing) was used, while custom CNN models were trained with an 80:10:10 ratio, based on their respective adaptation requirements. For VGG-16 and ResNet-50, both the 70:15:15 and 80:10:10 splits produced similar results. However, the 70:15:15 ratio was chosen because it allocated fewer resources to training, reducing computational demands while maintaining performance. In contrast, for the custom CNN models, the 80:10:10 ratio yielded better performance, as evidenced by higher accuracy and lower loss values compared to the 70:15:15 split, making it the preferred choice.

**Figure 10:**
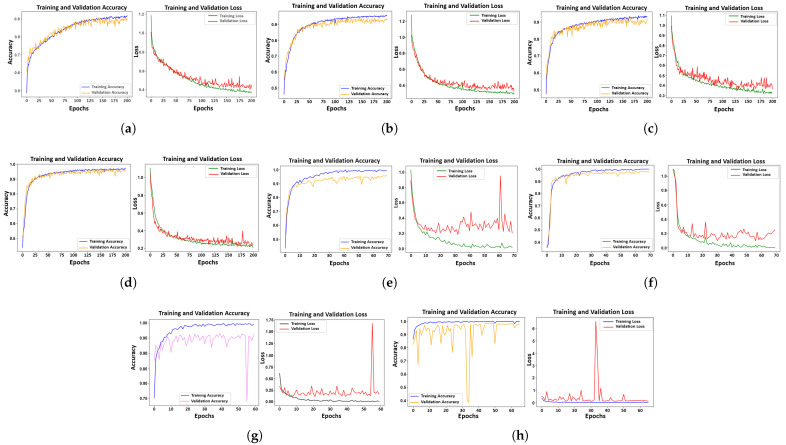
Accuracy and loss curve: (**a**) CNN_5_-128; (**b**) CNN_5_-240; (**c**) CNN_6_-128; (**d**) CNN_6_-240; (**e**) VGG-16_128_; (**f**) VGG-16_224_; (**g**) ResNet-50_128_; (**h**) ResNet-50_224_.

The experiments were conducted on a system configured with an Intel Core i7-11700 processor (8 cores, 16 threads, 2.50 GHz base frequency), 32 GB of DDR4 RAM, and running the Windows 10 operating system. The hardware was manufactured by Gigabyte Technology Co., Ltd., New Taipei City, Taiwan, featuring the B560M AORUS ELITE motherboard. The system designation is DESKTOP-J1TP30L, operating on a 64-bit architecture (x64-based PC). The deep learning models were trained using TensorFlow 2.x and NumPy 1.23.4 without GPU acceleration. The decision to use only the CPU was intentional, as the goal was to establish a resource-aware framework that could be deployed on systems without GPU acceleration, making it more accessible for practical applications. Consequently, some state-of-the-art algorithms, such as R-CNN, Mask R-CNN, and YOLO, were not feasible due to their high computational demands, which stem from their reliance on large-scale feature extraction and real-time processing capabilities. Figure 10 presents the training and loss curves for all models, illustrating their convergence behavior and performance during training.

As shown in the plots, the training accuracy and loss curves of all eight neural network models used in this study reveal key insights into their performance. The results demonstrate that higher image resolution improved model efficiency, emphasizing the importance of resolution in enhancing training outcomes. In general, all models displayed strong learning capacity, as reflected in their accuracy curves, with the exception of ResNet-50_128_ and ResNet-50_224_, which experienced some instability during the early stages of training.

However, this instability was not observed in the loss curves, where the validation curves for VGG-16_128_, VGG-16_224_, ResNet-50_128_, and ResNet-50_224_ struggled to converge, despite the models’ inherent robustness. This could be attributed to the input image dimensions, as the VGG-16_224_ and ResNet-50_224_ models showed greater stability than their 128 × 128 counterparts. Additionally, the high learning rate used due to the complexity of the dataset may have contributed to the observed fluctuations. While early fluctuations in accuracy and loss curves do not necessarily indicate poor performance, they suggest potential challenges in model convergence. Upon closer inspection, despite the fluctuations, ResNet-50_224_ demonstrated the ability to adapt and stabilize towards the end of the training process, showcasing its robustness and capacity to efficiently optimize and generalize from the data.

To provide a more comprehensive evaluation, the models were further assessed using the test dataset, and the results are summarized in Table 11 below.

In our assessment, macro-averaging was preferred over weighted averaging for the evaluation metrics to ensure that the models were evaluated based on their performance across each individual class. Given the slight imbalance in our dataset classes, macro-averaging was chosen to provide a more balanced evaluation. As shown in Table 11, VGG-16_224_ and ResNet-50_224_ outperformed the other models across various metrics. Specifically, ResNet-50_224_ achieved the best performance in most cases, including accuracy, loss, precision, recall, and F1-score. On the other hand, VGG-16_224_ excelled in specificity and mean average precision (mAP).

While ResNet-50_224_ can be considered the best classifier based on these results, it is important to note that specificity and mAP are also critical metrics. In particular, mAP is often the most important metric for evaluation, especially in multi-class and imbalanced data scenarios. Notably, ResNet-50_224_ did not achieve the highest mAP score, suggesting that other models, such as VGG-16_224_, may offer better performance in certain aspects.

To gain deeper insights into the models’ performance, the confusion matrix was also employed to evaluate the actual class classification performance, focusing on TP, FP, TN, and FN.

**Figure 11:**
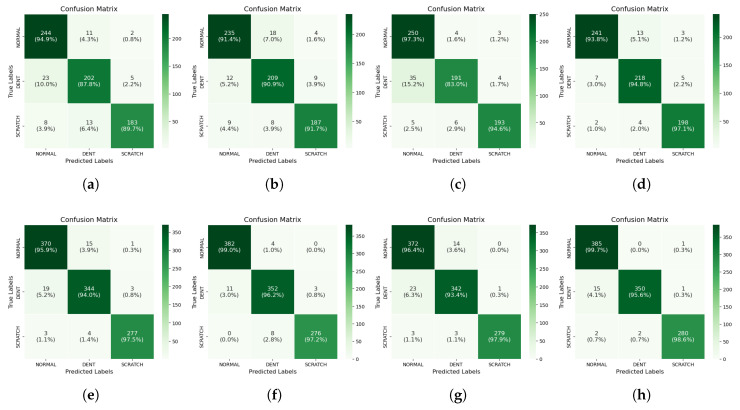
Confusion matrix: (**a**) CNN_5_-128; (**b**) CNN_5_-240; (**c**) CNN_6_-128; (**d**) CNN_6_-240; (**e**) VGG-16_128_; (**f**) VGG-16_224_; (**g**) ResNet-50_128_; (**h**) ResNet-50_224_.

Figure 11 displays the confusion matrix for all models. The reduced performance of most models can be attributed to the high FN values in the dent class, with many datasets incorrectly predicted as belonging to the normal class. Overall, the dent class had the highest FN, while the scratch class had the lowest FN on average across all models. The normal class consistently showed the highest percentage of TP, whereas the dent class had the lowest percentage of TP.

A comparative analysis of the performance of VGG-16_224_ and ResNet-50_224_—based on their confusion matrix evaluations shown in Figure 11f and Figure 11h, respectively—was conducted to determine the superior model, as these two emerged as the top performers. The performance of each model across all classes was evaluated to identify their respective strengths and weaknesses in class prediction. ResNet-50_224_ demonstrated superior TP performance for the normal and scratch classes, while VGG-16_224_ excelled in the dent class. Furthermore, ResNet-50_224_ achieved lower FN scores in two classes (normal and scratch), while VGG-16_224_ recorded a lower FN only for the dent class. A similar pattern was observed in the FP assessment, where ResNet-50_224_ exhibited lower scores for the dent and scratch classes, whereas VGG-16_224_ had a lower score for the normal class.

These findings suggest that ResNet-50_224_ demonstrated greater data adaptability and overall efficiency compared to VGG-16_224_ in this study.

### 5.1. Computation Efficiency Evaluation

One of the key factors in determining an optimal model is its computational efficiency. Therefore, this section discusses the selection of an adequate model and offers suggestions for alternative models that could be considered when computational efficiency is a critical factor.

For a more in-depth analysis, floating point operations (FLOPs) are introduced to concisely determine the computational energy requirements of each model. FLOPs are a key metric for assessing model complexity, especially in deep learning models. They provide valuable insights into the computational requirement of a model, which is key for model optimization, hardware implementation, and real-time performance.

**Figure 12:**
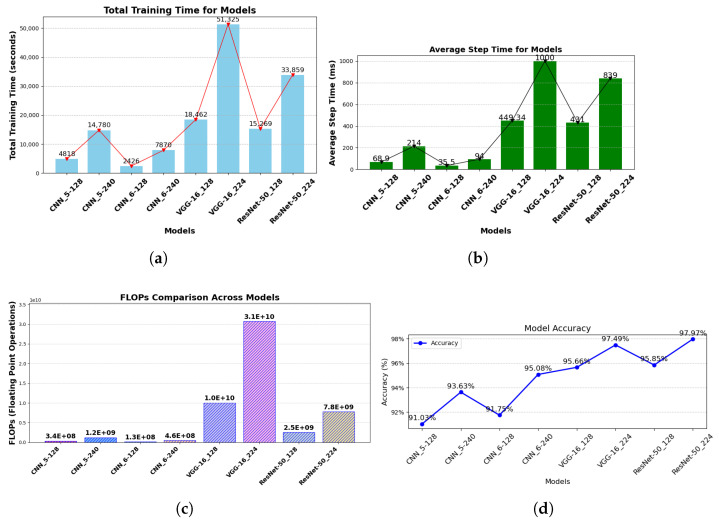
(**a**) Models’ total training time; (**b**) Models’ average training step for one image data; (**c**) Models’ floating points operations; (**d**) Models’ accuracy.

Figure 12a–c provide a comprehensive computational analysis of the models implemented in this study. The plots clearly show that the custom CNN models outperformed the pre-trained models (VGG-16 and ResNet-50 variants) in terms of computational efficiency. An interesting observation from the computational analysis is that the CNN_5_ models exhibited higher computational energy demands for their respective input pixel dimensions, despite having shallower architectures compared to the CNN_6_ models. This behavior may be attributed to the complexity of the dataset, which likely challenges the CNN_5_ architectures in fully capturing and learning its features.

While ResNet-50_224_ demonstrated exceptional performance across various evaluation metrics, its significant computational energy demand raises concerns for applications where computational efficiency is a key factor, as shown in Figure 12a,b. Furthermore, Figure 12c, which displays the FLOPs of the models, revealed similar findings. ResNet-50_224_—with a total of 7,751,510,674 FLOPs—may not be the ideal choice for real-time system applications due to its computational complexity. In contrast, CNN_6_-240 presents itself as a more desirable option. Its accuracy—reaching up to 95% as shown in Figure 12d—though lower than that of ResNet-50_224_, requires significantly less computational energy and has FLOPs of 459,072,914, suggesting suitability for real-time system implementation. This can be attributed to its lighter architecture, making it a practical alternative despite its slightly lower accuracy compared to ResNet-50_224_. Moreover, its computational efficiency offers an opportunity for further hyperparameter tuning, which could enhance its accuracy. This makes the CNN_6_-240 model adaptable, cost-effective, and well-suited for resource-sensitive environments without compromising performance.

### 5.2. K-Fold Model Evaluation

To validate the two best-performing models—ResNet-50_224_ (best overall) and CNN_6_-240 (most computationally efficient)—we implemented a 3-fold cross-validation. This technique partitions the dataset into three folds, using two folds for training and one for validation in each iteration. By rotating the validation fold, we ensure every data point contributes to both training and evaluation, reducing bias from a single split, hence providing a more generalized performance assessment. Stratified sampling was implemented to ensure robust evaluation.

**Figure 13:**
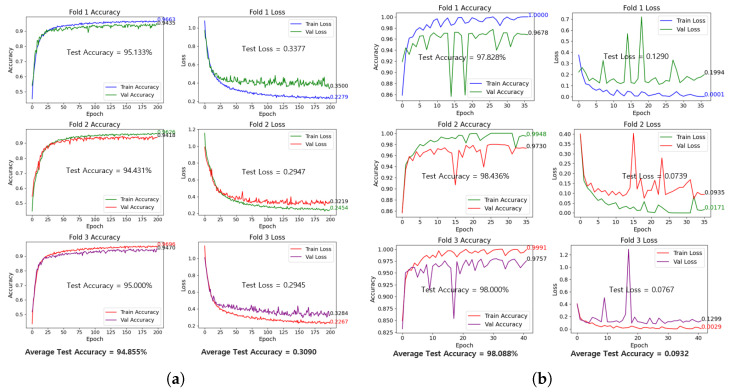
3-Fold validation curves: (**a**) CNN_6_-240; (**b**) ResNet-50_224_.

Figure 13 illustrate the outcomes of the 3-fold cross-validation, showing both models’ accuracy and loss curves, alongside their respective test accuracies and losses. CNN_6_-240 maintained an average k-fold test accuracy of 94.86%, closely mirroring its initial test accuracy of 95.08%. This reflects its strong efficiency and reliability in maintaining performance across different splits. Meanwhile, ResNet-50_224_ demonstrated superior accuracy, achieving an average of 98.088% with a reduced average loss of 0.0932, outperforming its original test results of 97.97% accuracy and 0.1030 loss. These findings highlight ResNet-50_224_’s advantage in generalization and accuracy, while CNN_6_-240 remains an effective, computationally efficient alternative.

## 6. Conclusions and Further Works

In this study, we proposed a framework for image data generation and fault classification on glossy and curved surfaces. Data collection was achieved using two complementary techniques: a Basler vision camera with specialized lighting to capture front-view images of heart case glossy surface samples while minimizing reflectiveness, and a laser displacement sensor paired with a robotic arm to accurately capture the curved surfaces of the samples.

Our dataset—consisting of three classes (HCC, DCC, and SCC)—was used to train eight deep neural networks. These networks were selected for their adaptability to the data and lower computational energy requirements. The architectures included four custom traditional CNNs (CNN_5_-128, CNN_5_-240, CNN_6_-128, CNN_6_-240) trained on image dimensions of 128 × 128 and 240 × 240 pixels, as well as four pre-established models: two variations of VGG-16 (VGG-16_128_, VGG-16_224_) and two variations of ResNet-50 (ResNet-50_128_, ResNet-50_224_), which were trained on images of 128 × 128 and 224 × 224 pixels, respectively. From our evaluation results, ResNet-50_224_ achieved the highest accuracy at 97.97%, followed closely by VGG-16_224_ with an accuracy of 97.49%. However, ResNet-50_224_’s computational demands were less efficient compared to some of the custom traditional CNN architectures. Notably, CNN_6_-240 delivered an accuracy of 95.08% with an average step time of 94 milliseconds, compared to ResNet-50_224_’s 839 milliseconds, making CNN_6_-240 a viable choice in scenarios where computational efficiency is paramount.

Our framework significantly advances glossy and curved surface defect detection by integrating specialized imaging hardware with tailored deep learning models. Using a Basler camera with custom lighting minimizes reflections on glossy surfaces, while a laser displacement sensor with a robotic arm precisely captures curved surfaces—yielding high-quality, reliable data. Beyond technical metrics, our framework also has significant industrial implications. Its deployment in manufacturing could lead to substantial cost savings by reducing defect rates and minimizing downtime, ultimately improving product quality and competitiveness.

For future work, we plan to further optimize and enhance the custom CNN models to achieve higher accuracy while reducing energy consumption. The lag observed in most models can likely be attributed to the high FN rate of the dent class, as indicated by the confusion matrix. This issue is more likely caused by feature overlap with normal surfaces rather than class imbalance. To address this, targeted data augmentation like CLACHE (Contrast Limited Adaptive Histogram Equalization), feature engineering, and loss function modifications can be employed to enhance detection accuracy. In addition, systematic optimization techniques such as grid search and Bayesian optimization can be introduced to fine-tune hyperparameters for improved model performance. Furthermore, we aim to explore federated learning for multi-factory deployment, enabling collaborative, privacy-preserving model training across distributed production sites. We also plan to investigate lightweight model distillation techniques to facilitate real-time, energy-efficient deployment in industrial settings, potentially achievable through fine-tuning the custom CNN.

However, a key limitation of our approach is its reliance on controlled lighting conditions and specific robotic arm configurations. The reliance on controlled lighting conditions may limit the framework’s applicability in environments with no lighting, variable lighting, or uncontrolled lighting, such as outdoor or poorly lit industrial settings. For example, in environments with uncontrolled lighting, the system’s accuracy could drop significantly due to increased reflections and noise. Furthermore, the use of a robotic arm for precise positioning introduces scalability limitations and challenges in integrating with other automated systems. Additionally, its deployment is heavily dependent on expert knowledge, which may restrict widespread adoption. Future work will focus on mitigating these limitations by improving adaptability to diverse lighting conditions and exploring alternative data acquisition methods.

## Figures and Tables

**Table 1:** Camera Specification.

Camera Specification Details	
Product Name	Hikvision MV-CA050-20UM
Lens	f 35 mm
Working Distance	20 mm
Pixel	5 MP
Field of view (FOV)	91 mm × 72 mm
Manufacturer	Hangzhou Hikvision Digital Technology Co., Ltd., headquartered in Hangzhou, Zhejiang, China.

**Table 2:** Lighting specification.

Lighting Specification Details	
Product Name	surf.Finder-SF
Dimension	335 × 325 × 330 mm^3^
(FOV)	60 × 60 mm^2^
Weight	6.9 kg
Other Accessories	Built-in Controller, S/W Package (SDK, Viewer), Lan cable, SMPS, Dongle key
Manufacturer	Didim Sensor Co., Ltd. Seoul, South Korea.

**Table 3:** Laser displacement specification.

Laser Specification Details	
Product Name	LJX-8080
Measuring range Z-axis (height)	73 mm (±20.5 mm)
(Measuring range X-axis (width))	35.0 mm
Repeatability Z-axis (height)	0.5 μ m
Repeatability X-axis (width)	1.0 μ m

**Table 4:** Measurement results for shooting from the side.

Number of Inspections	Number of Coordinates Moved	Time (s)	Inspection Speed (mm/s)
1	22	21.2	11.5
2	23	20.8	11.5
3	23	23.4	11.5
4	24	25.1	11.5
5	23	24.2	11.5
6	23	25.1	11.5
7	23	23.1	11.5
8	23	22.9	11.5
9	23	22.9	11.5
10	23	26.0	11.5

**Table 5:** Selected path information when shooting from the side.

Number of Coordinates Moved	Measurement Time (s)	Inspection Speed (mm/s)
23	18.2	11.5

**Table 6:** Selected path information while shooting from the side.

Number of Coordinates Moved	Measurement Time (s)	Inspection Speed (mm/s)
23	14.6	13.2

**Table 7:** Summary of different classes of images involved in the study.

Label	Image Class	Description
HCC	Healthy-case class	The healthy cases of the image samples.
DCC	Dented-case class	The dented cases of the image samples.
SCC	Scratched-case class	The scratched cases of the image samples.

**Table 8:** Summary of the models used in the study.

Model Name	Input Image Resolution	Number of Layers	Description
CNN_5_-128	128 × 128	Five (5)	A traditional CNN model with 128 × 128 image input dimension and five layers.
CNN_5_-240	240 × 240	Five (5)	A traditional CNN model with 240 × 240 image input dimension and five layers
CNN_6_-128	128 × 128	Six (6)	A traditional CNN model with 128 × 128 image input dimension and six layers
CNN_6_-240	240 × 240	Six (6)	A traditional CNN model with 240 × 240 image input dimension and six layers
VGG-16_128_	128 × 128	Default with one dense layer	A typical VGG-16 algorithm with 128 × 128 image input dimension and a dense layer
VGG-16_224_	224 × 224	Default with one dense layer	A typical VGG-16 algorithm with 224 × 224 image input dimension and a dense layer
ResNet-50_128_	128 × 128	Default with one dense layer	A typical ResNet-50 algorithm with 128 × 128 image input dimension and a dense layer
ResNet-50_224_	224 × 224	Default with one dense layer	A typical ResNet-50 algorithm with 224 × 224 image input dimension and a dense layer

**Table 9:** Architectures of the custom CNN models.

Model Name	Number of Conv Layers/Stride	Kernel Sizes/Learning Rate	Pooling Layer/Size	Activation Functions	Fully Connected Layers/Dropout	Image Dimension	Batch Size/Epochs
CNN_5_-128	3/1	3 × 3/0.001	3/2 × 2	Relu/Softmax	2/0.1	128 × 128	16/200
CNN_5_-240	3/1	3 × 3/0.001	3/2 × 2	Relu/Softmax	2/0.1	240 × 240	16/200
CNN_6_-128	4/1	3 × 3/0.001	3/2 × 2	Relu/Softmax	2/0.1	128 × 128	16/200
CNN_6_-240	4/1	3 × 3/0.001	3/2 × 2	Relu/Softmax	2/0.1	240 × 240	16/200

**Table 10:** Summary of the modified VGG-16 and ResNet-50 architecture employed in the study.

Model Name	Pre-Trained VGG-16 Model	Image Dimension	Dense Layer	Dropout Rate	Output Layer	Optimizer/Learning Rate
VGG-16_128_	ImageNet weights	128 × 128	256, ReLU	0.2	Softmax	Adam/0.0001
VGG-16_224_	ImageNet weights	224 × 224	256, ReLU	0.2	Softmax	Adam/0.0001
ResNet-50_128_	ImageNet weights	128 × 128	256, ReLU	0.2	Softmax	Adam/0.0001
ResNet-50_224_	ImageNet weights	224 × 224	256, ReLU	0.2	Softmax	Adam/0.0001

**Table 11:** Performance Eevaluation summary.

Model	Test Accuracy	Test Loss	Precision	Recall	F1 Score	Specificity	mAP
CNN_5_-128	91.03	0.4064	91.47	90.82	91.07	95.30	0.9697
CNN_5_-240	93.63	0.3651	91.41	91.33	91.36	95.62	0.9723
CNN_6_-128	91.75	0.3459	92.58	91.64	91.86	95.72	0.9795
CNN_6_-240	95.08	0.2753	95.09	95.21	95.14	97.53	0.9878
VGG-16_128_	95.66	0.1981	95.91	95.79	95.85	97.75	0.9906
VGG-16_224_	97.49	0.1911	97.61	97.61	97.52	98.71	0.9963
ResNet-50_128_	95.85	0.2073	96.21	96.21	96.12	97.83	0.9869
ResNet-50_224_	97.97	0.1030	98.16	97.99	98.05	97.78	0.9942

## Data Availability

The data presented in this study are available upon request from the corresponding author. The data are not publicly available due to laboratory regulations.

## References

[1] Lee J.-H., Okwuosa C.N., Shin B.C., Hur J.-W. (2024). A Spectral-Based Blade Fault Detection in Shot Blast Machines with XGBoost and Feature Importance. J. Sens. Actuator Netw..

[2] Zhou L., Zhang L., Konz N. (2023). Computer Vision Techniques in Manufacturing. IEEE Trans. Syst. Man Cybern. Syst..

[3] Zhou L., Zhang L., Konz N., Balamuralidhar N., Tilon S., Nex F. (2021). MultEYE: Monitoring System for Real-Time Vehicle Detection, Tracking and Speed Estimation from UAV Imagery on Edge-Computing Platforms. Remote Sens..

[4] Islam M.R., Zamil M.Z.H., Rayed M.E., Kabir M.M., Mridha M.F., Nishimura S. (2024). Deep Learning and Computer Vision Techniques for Enhanced Quality Control in Manufacturing Processes. IEEE Access.

[5] Chen Y., Ding Y., Zhao F., Zhang E., Wu Z., Shao L. (2021). Surface Defect Detection Methods for Industrial Products: A Review. Appl. Sci..

[6] Zhou A., Zhang M., Li M., Shao W. Defect Inspection Algorithm of Metal Surface Based on Machine Vision. Proceedings of the 2020 12th International Conference on Measuring Technology and Mechatronics Automation (ICMTMA).

[7] Sun H., Teo W.-T., Wong K., Dong B., Polzer J., Xu X. (2024). Automating Quality Control on a Shoestring, a Case Study. Machines.

[8] Chin R.T., Harlow C.A. (1982). Automated Visual Inspection: A Survey. IEEE Trans. Pattern Anal. Mach. Intell..

[9] Abd Al Rahman M., Mousavi A. (2020). A Review and Analysis of Automatic Optical Inspection and Quality Monitoring Methods in Electronics Industry. IEEE Access.

[10] Tang Y., Sun K., Zhao D., Lu Y., Jiang J., Chen H. Industrial Defect Detection Through Computer Vision: A Survey. Proceedings of the 2022 7th IEEE International Conference on Data Science in Cyberspace (DSC).

[11] Tzampazaki M., Zografos C., Vrochidou E., Papakostas G.A. (2024). Machine Vision—Moving from Industry 4.0 to Industry 5.0. Appl. Sci..

[12] Trivedi C., Bhattacharya P., Prasad V.K., Patel V., Singh A., Tanwar S., Sharma R., Aluvala S., Pau G., Sharma G. (2024). Explainable AI for Industry 5.0: Vision, Architecture, and Potential Directions. IEEE Open J. Ind. Appl..

[13] Jian Z., Wang X., Zhang X., Su R., Ren M., Zhu L. (2024). Task-Specific Near-Field Photometric Stereo for Measuring Metal Surface Texture. IEEE Trans. Ind. Inform..

[14] Heckbert P.S. (1986). Survey of Texture Mapping. IEEE Comput. Graph. Appl..

[15] Foster P., Johnson C., Kuipers B. The Reflectance Field Map: Mapping Glass and Specular Surfaces in Dynamic Environments. Proceedings of the 2023 IEEE International Conference on Robotics and Automation (ICRA).

[16] Müller V., Buxton B., Cipolla R. (1996). Elimination of specular surface-reflectance using polarized and unpolarized light. Computer Vision—ECCV ’96. ECCV 1996.

[17] Yoon K., Seol J., Kim K.G. (2022). Removal of Specular Reflection Using Angle Adjustment of Linear Polarized Filter in Medical Imaging Diagnosis. Diagnostics.

[18] Kaminokado T., Iwai D., Sato K. Augmented Environment Mapping for Appearance Editing of Glossy Surfaces. Proceedings of the 2019 IEEE International Symposium on Mixed and Augmented Reality (ISMAR).

[19] Yan T., Li H., Gao J., Wu Z., Lau R.W.H. (2023). Single Image Reflection Removal From Glass Surfaces via Multi-Scale Reflection Detection. IEEE Trans. Consum. Electron..

[20] Sudo H., Yukushige S., Muramatsu S., Inagaki K., Chugo D., Hashimoto H. Detection of Glass Surface Using Reflection Characteristic. Proceedings of the IECON 2021—47th Annual Conference of the IEEE Industrial Electronics Society.

[21] Yan T., Gao J., Xu K., Zhu X., Huang H., Li H., Wah B., Lau R.W. (2025). GhostingNet: A Novel Approach for Glass Surface Detection With Ghosting Cues. IEEE Trans. Pattern Anal. Mach. Intell..

[22] Yuan S., Li L., Chen H., Li X. (2023). Surface Defect Detection of Highly Reflective Leather Based on Dual-Mask-Guided Deep-Learning Model. IEEE Trans. Instrum. Meas..

[23] Jiang Q., Tan D., Li Y., Ji S., Cai C., Zheng Q. (2020). Object Detection and Classification of Metal Polishing Shaft Surface Defects Based on Convolutional Neural Network Deep Learning. Appl. Sci..

[24] Wang Y., Xu Q., Li Y., Wei C., Wei L. (2024). 3-D *μ*XRF Imaging System for Curved Surface. IEEE Trans. Instrum. Meas..

[25] Huo S., Navarro-Alarcon D., Chik D. (2020). A Robotic Line Scan System with Adaptive ROI for Inspection of Defects over Convex Free-form Specular Surfaces. arXiv.

[26] Rao L.J., Ramkumar M., Kothapalli C., Savarapu P.R., Basha C.Z. Advanced computerized Classification of X-ray Images using CNN. Proceedings of the 2020 Third International Conference on Smart Systems and Inventive Technology (ICSSIT).

[27] Nidhya R., Kalpana R., Smilarubavathy G., Keerthana S.M. Brain Tumor Diagnosis with MCNN-Based MRI Image Analysis. Proceedings of the 2023 1st International Conference on Optimization Techniques for Learning (ICOTL).

[28] Srinivasan S., Francis D., Mathivanan S.K., Rajadurai H., Shivahare B.D., Shah M.A. (2024). A hybrid deep CNN model for brain tumor image multi-classification. BMC Med. Imaging.

[29] Rajeshkumar C., Soundar K.R., Sneha M., Maheswari S.S., Lakshmi M.S., Priyanka R. Convolutional Neural Networks (CNN) based Brain Tumor Detection in MRI Images. Proceedings of the 2023 5th International Conference on Smart Systems and Inventive Technology (ICSSIT).

[30] García-Navarrete O.L., Correa-Guimaraes A., Navas-Gracia L.M. (2024). Application of Convolutional Neural Networks in Weed Detection and Identification: A Systematic Review. Agriculture.

[31] Fei X., Wu S., Miao J., Wang G., Sun L. (2024). Lightweight-VGG: A Fast Deep Learning Architecture Based on Dimensionality Reduction and Nonlinear Enhancement for Hyperspectral Image Classification. Remote Sens..

[32] He K., Zhang X., Ren S., Sun J., Sun L. Deep Residual Learning for Image Recognition. Proceedings of the 2016 IEEE Conference on Computer Vision and Pattern Recognition (CVPR).

[33] Shah S.R., Qadri S., Bibi H., Shah S.M.W., Sharif M.I., Marinello F. (2023). Comparing Inception V3, VGG 16, VGG 19, CNN, and ResNet 50: A Case Study on Early Detection of a Rice Disease. Agronomy.

[34] Sahin M.E., Ulutas H., Yuce E., Erkoc M.F. (2023). Detection and classification of COVID-19 by using faster R-CNN and mask R-CNN on CT images. Neural Comput. Appl..

[35] Wang X., Sun L., Lu C., Li B. (2024). A Novel Transformer Network with a CNN-Enhanced Cross-Attention Mechanism for Hyperspectral Image Classification. Remote Sens..

[36] He J., Lv F., Liu J., Wu M., Chen B., Wang S. (2025). C2T-HR3D: Cross-Fusion of CNN and Transformer for High-Speed Railway Dropper Defect Detection. IEEE Trans. Instrum. Meas..

[37] Taye M.M. (2023). Theoretical Understanding of Convolutional Neural Network: Concepts, Architectures, Applications, Future Directions. Computation.

[38] Li Z., Liu F., Yang W., Peng S., Zhou J. (2022). A Survey of Convolutional Neural Networks: Analysis, Applications, and Prospects. IEEE Trans. Neural Netw. Learn. Syst..

[39] Sa-Couto L., Wichert A. (2021). Simple Convolutional-Based Models: Are They Learning the Task or the Data?. Computation.

[40] Ige A.O., Sibiya M. (2023). State-of-the-Art in 1D Convolutional Neural Networks: A Survey. IEEE Access.

[41] Liu J., Wang T., Skidmore A., Sun Y., Jia P., Zhang K. (2023). Integrated 1D, 2D, and 3D CNNs Enable Robust and Efficient Land Cover Classification from Hyperspectral Imagery. Remote Sens..

[42] Singh S.P., Wang L., Gupta S., Goli H., Padmanabhan P., Gulyás B. (2020). 3D Deep Learning on Medical Images: A Review. Remote Sens..

[43] Krichen M. (2023). Convolutional Neural Networks: A Survey. Computers.

[44] Buslaev A., Iglovikov V.I., Khvedchenya E., Parinov A., Druzhinin M., Kalinin A.A. (2020). Albumentations: Fast and Flexible Image Augmentations. Information.

[45] Cong S., Zhou Y. (2023). A review of convolutional neural network architectures and their optimizations. Artif. Intell. Rev..

[46] Toennies K.D. (2024). Feature Extraction by Convolutional Neural Network. An Introduction to Image Classification.

[47] Chen L., Li S., Bai Q., Yang J., Jiang S., Miao Y. (2021). Review of Image Classification Algorithms Based on Convolutional Neural Networks. Remote Sens..

[48] Galanis N.-I., Vafiadis P., Mirzaev K.-G., Papakostas G.A. (2022). Convolutional Neural Networks: A Roundup and Benchmark of Their Pooling Layer Variants. Algorithms.

[49] Jeczmionek E., Kowalski P.A. (2021). Flattening Layer Pruning in Convolutional Neural Networks. Symmetry.

[50] Sarıateş M., Özbay E. (2025). A Classifier Model Using Fine-Tuned Convolutional Neural Network and Transfer Learning Approaches for Prostate Cancer Detection. Appl. Sci..

[51] Maniatopoulos A., Mitianoudis N. (2021). Learnable Leaky ReLU (LeLeLU): An Alternative Accuracy-Optimized Activation Function. Information.

[52] Kulathunga N., Ranasinghe N.R., Vrinceanu D., Kinsman Z., Huang L., Wang Y. (2021). Effects of Nonlinearity and Network Architecture on the Performance of Supervised Neural Networks. Algorithms.

[53] Solovyeva E., Abdullah A. (2021). Binary and Multiclass Text Classification by Means of Separable Convolutional Neural Network. Inventions.

[54] Salehin I., Kang D.-K. (2023). A Review on Dropout Regularization Approaches for Deep Neural Networks within the Scholarly Domain. Electronics.

[55] Simonyan K., Zisserman A. (2015). Very Deep Convolutional Networks for Large-Scale Image Recognition. arXiv.

[56] Balderas L., Lastra M., Benítez J.M. (2024). Optimizing Convolutional Neural Network Architectures. Mathematics.

[57] Jiang W., Han H., Zhang Y., Wang J., He M., Gu W., Mu J., Cheng X. (2024). Graph Neural Networks for Routing Optimization: Challenges and Opportunities. Sustainability.

[58] Kim S.P., Cho H.S., Hong S.H., Cho H.J., Sohn H.G. Genetic algorithm to find the shortest path on raster data. Proceedings of the 2014 International Conference on Control, Automation and Information Sciences (ICCAIS 2014).

